# Application of failure mode and effects analysis to validate a novel hybrid Linac QC program that integrates automated and conventional QC testing

**DOI:** 10.1002/acm2.13798

**Published:** 2022-12-01

**Authors:** Michael Pearson, Victoria Butterworth, Sarah Misson‐Yates, Marium Naeem, Regina Gonzalez Vaz, David Eaton, Tony Greener

**Affiliations:** ^1^ Medical Physics Department Guy's and St Thomas' Hospital London UK; ^2^ School of Biomedical Engineering & Imaging Sciences King's College London London UK

**Keywords:** FMEA, Linac quality control (QC), linear accelerator, machine performance check (MPC), quality assurance

## Abstract

A hybrid quality control (QC) program was developed that integrates automated and conventional Linac QC, realizing the benefits of both automated and conventional QC, increasing efficiency and maintaining independent measurement methods. Failure mode and effects analysis (FMEA) was then applied in order to validate the program prior to clinical implementation. The hybrid QC program consists of automated QC with machine performance check and DailyQA3 array on the TrueBeam Linac, and Delta4 volumetric modulated arc therapy (VMAT) standard plan measurements, alongside conventional monthly QC at a reduced frequency. The FMEA followed the method outlined in TG‐100. Process maps were created for each treatment type at our center: VMAT, stereotactic body radiotherapy (SBRT), conformal, and palliative. Possible failure modes were established by evaluating each stage in the process map. The FMEA followed semiquantitative methods, using data from our QC records from eight Linacs over 3 years for the occurrence estimates, and simulation of failure modes in the treatment planning system, with scoring surveys for severity and detectability. The risk priority number (RPN) was calculated from the product of the occurrence, severity, and detectability scores and then normalized to the maximum and ranked to determine the most critical failure modes. The highest normalized RPN values (100, 90) were found to be for MLC position dynamic for both VMAT and SBRT treatments. The next highest score was 35 for beam position for SBRT, and the majority of scores were less than 20. Overall, these RPN scores for the hybrid Linac QC program indicated that it would be acceptable, but the high RPN score associated with the dynamic MLC failure mode indicates that it would be valuable to perform more rigorous testing of the MLC. The FMEA proved to be a useful tool in validating hybrid QC.

## 1 INTRODUCTION

In recent years, linear accelerator (Linac) manufactures have developed automated quality control (QC) tools that can be run daily as part of the morning run‐up routine. These include machine performance check (MPC) on the Varian TrueBeam and Halcyon Linacs (Varian Medical Systems, Inc., Palo Alto, CA, USA), the Essential TQA and Advanced TQA modules on TomoTherapy and Radixact (TomoTherapy, Inc., Madison, WI, USA), and the Elekta (Elekta AB, Stockholm, Sweden) Automated Quality Assurance (AQUA) tool. These systems generally run an automated sequence of kV and/or MV acquisitions at a range of set machine parameters and compare results versus the baseline acquisition. They have been shown to have high accuracy^1^, ^2^, ^3^, ^4^, ^5^, ^6^, ^7^, ^8^, ^9^ and are powerful because multiple QC tests are carried out on a daily basis, testing parameters that would normally be performed monthly as part of a conventional QC program.

There are various possible approaches that can be taken when integrating these tools into a Linac QC program in the clinic. They may be used complementary to conventional testing, or to replace conventional QC.^2^, ^6^ At our center, having established our confidence in MPC through the use and testing of MPC over 3 years on eight Linacs, we have integrated MPC into our conventional QC program, utilizing the hybrid QC approach.^7^ Rather than the complete replacement of conventional QC testing with automated MPC tests, the hybrid QC approach is a hybrid of automated and conventional QC methods. Where particular test parameters are tested as part of the daily automated MPC, the frequency of corresponding conventional monthly QC tests is reduced to quarterly or annual, as well as being performed in response to specific faults and repairs.

Failure mode and effects analysis (FMEA) has been carried out previously in relation to Linac QC^10^, ^11^, ^12^ following the methodology outlined in AAPM Task Group 100.^13^ The AAPM Medical Physics Practice Guideline 8.a.^10^ applied the FMEA risk assessment methodology to the Linac QC tests outlined in TG‐142^14^ in order to determine the most critical tests. Scoring was carried out by committee members from a range of institutions across the United States based on their local experience. This ensured that a wide range of radiotherapy treatment type and technology, age of equipment, and patient populations were represented in the scoring. This made their conclusions on test priority relevant to general radiotherapy delivery, but not necessarily directly relevant to an individual institution. FMEA has since been utilized to determine clinic‐specific test frequencies to maximize efficiency and patient treatment quality.^11^, ^12^ Efficiency savings were realized by reducing the testing frequency for the tests with low‐risk priority number (RPN) ranking.

In the TG‐100 methodology, scorers determine their estimates of each failure mode score based on their expertise and experience. O'Daniel et al.^11^ and Bonfantini et al.^12^ developed the methodology, employing quantitative^11^ and semiquantitative^12^ approaches to estimate the failure mode scores for Linac QC. Their occurrence scores were determined from the frequency of out of tolerance results in QC records, and severity scores were estimated by simulating errors in the treatment planning system, or from studies published in literature. In addition to applying FMEA specific to their clinic, Bonfantini et al.^12^ further applied it specific to each Linac in their center, identifying failure modes based on the particular treatments types on each Linac.

The previous application of FMEA has been to existing Linac QC programs, with the aim to identify critical tests and to make time‐saving efficiencies by reducing the frequencies of lower priority tests. The aim of this work is to apply FMEA to the newly developed hybrid QC program, in order to validate this new approach to Linac QC. We developed process maps for each type of patient treatment on our Linacs and used these to identify possible failure modes at each stage. The FMEA followed semiquantitative methods, using data from our QC records from eight Linacs over 3 years for the occurrence estimates, alongside scoring surveys for severity and detectability, with simulated faults in the treatment planning system on a sample of test patients informing the severity estimates.

## 2 MATERIALS AND METHODS

### 2.1 Hybrid QC

The hybrid QC program that we developed for our department is outlined in Table 1. The program integrates automated and conventional testing, with the aim of capitalizing on the benefits of both approaches. The key automated elements are MPC and DailyQA3 (Sun Nuclear Corporation, Melbourne, FL, USA). MPC measures beam output, uniformity, and various geometric parameters with test tolerances less than or equivalent to those recommended by TG‐142 and IPEM Report 81v2. The efficiency of the MPC automated testing allows the daily monitoring of many parameters, some of which may have only be tested on a monthly basis before in a conventional QC program. This offers the possibility of detecting faults earlier instead of in the next monthly QC, as well as the ability to observe daily trends. In addition to this, we have weekly DailyQA3 array measurements and monthly volumetric modulated arc therapy (VMAT) standard plan measurements on the Delta4 (ScandiDos AB, Uppsala, Sweden). The DailyQA3 is a daily QC constancy check device with a number of ionization chambers and diode detectors to measure output constancy, flatness, symmetry, energy, and radiation field size.^16^ The MPC and DailyQA3 provide similar levels of automation. Both require the initial manual aspect of setting up the MPC phantom or DailyQA3 device on the treatment couch. After this, MPC requires the operator to mode up to drive the machine to the initial position and then initiate the beam for each beam energy. The subsequent acquisition and analysis is fully automated. We have implemented a standard plan in order to automate the process with the DailyQA3 to a similar extent. The standard plan consists of a sequence of fields with preset machine parameters for each beam energy and is delivered in Machine QA mode, enabling automated machine setup, and then analysis of each measured beam with the DailyQA3 application. Both MPC and DailyQA3 utilize a traffic light system to indicate to the operator the outcome of the testing. If desired, for example, in the case of a failing parameter, the operator can interrogate the results for detail on the specific parameters that have failed and review the trends of previous test results.

**TABLE 1:** The test frequencies, the type of testing (automated or conventional), and the action level and suspend level tolerances for the tests in the hybrid quality control (QC) program

Frequency	QC type	Test	Action	Suspend
Daily	Conventional Automated	Laser congruence ODI 2D–2D match (imaging isocenter/couch move) MPC ‐ Output constancy, uniformity, beam position, collimator angle, jaw size, MLC position, couch movement scales, gantry angle, kV and MV imager isocenter off set, isocenter size	1 mm 2 mm 1 mm	2 mm 3 mm 2 mm
Weekly	Automated	DailyQA3 ‐ Output constancy, flatness, symmetry, energy, and radiation field size		
Monthly	Conventional	Output Wedge factor Sweeping gap VMAT MLC Picket Fence VMAT Delta4 Standard Plan Isocenter Verification	1.5% 1.5% 2% (1%^a^) ≥95% pass^b^ 0.5 mm	2% 2% 3% (1.5%^a^) 1 mm ≥90% pass^b^ 1 mm
Quarterly	Conventional	Cross hair Light field Jaw size Lasers vs. optical–mechanical isocenter ODI vs. optical–mechanical isocenter Flatness and symmetry kV and CBCT image quality	0.5 mm 2%	1 mm 1 or 2 mm^c^ 1 mm 2 mm 3%
Annual	Conventional	All other recommended monthly, quarterly, semiannual and annual tests in Report 81v2 and TG‐142 with test frequency ≥ monthly		

Abbreviations: MPC, machine performance check; VMAT, volumetric modulated arc therapy.

^a^STx Linac tolerance is 1% and 1.5%.

^b^3%, 3 mm local gamma test.

^c^1 mm jaw setting of ≤15 cm, 2 mm for 20 cm.

Rather than full reliance on these automated tests, and complete replacement of the conventional QC tests that are performed by MPC and DailyQA3, we have adopted a hybrid model, where we have reduced the frequency of the corresponding conventional QC testing to quarterly or annual. The hybrid QC approach reaps the benefits of automated QC of reduced setup and operator error, increased measurement accuracy and time‐savings. The conventional QC testing at reduced frequency maintains independent QC testing of the Linacs, provides an ongoing independent check of MPC and DailyQA3, and maintains medical physicist QC skills. Our procedure in cases of faults or repairs is to perform both MPC and conventional QC, believing it essential to perform independent testing prior to returning the Linac to clinical use. The time‐saving, in both Linac and Medical Physicist time, equates at least 2 h per monthly QC session per Linac. We believe the concept of hybrid QC is not exclusive to the use of MPC and DailyQA3 with conventional QC but could equally be applied to any situation where automated Linac QC tools are utilized. This could be with another Linac vendor's QC tools, in‐house developed QC, or automated tools from dosimetry equipment manufacturers such as SunCHECK Machine (Sun Nuclear Corporation, Melbourne, FL, USA), which automates imaging, MLC, and VMAT QC. The concept behind the hybrid QC program is that both automated and conventional QC is integrated into the QC program, to receive the benefits of both systems, but maintain independence through performing conventional QC tests at a lower frequency.

Alongside MPC, the daily testing in our hybrid QC program consists of some standard conventional daily QC tests as recommended by TG‐142^14^ and IPEM Report 81v2.^15^ The weekly testing is performed with DailyQA3, which has test parameters that deliberately overlap with the MPC testing to provide regular independent checks. The daily and weekly checks are performed by therapists and are reviewed by physicists on a monthly basis, with any daily out of tolerance results communicated immediately.

We measure standard plans on the Delta4 each month in order to perform a full system QC for VMAT delivery. Here, we check that gamma pass rates are consistent for a 6 MV nasopharynx plan and a 10 MV prostate and nodes plan, confirming the consistency of the dose rate, MLC, and gantry speed control. These plans were chosen due to their complexity, each having wide ranges in dose rate and complex MLC pattern of delivery, as well as using nearly the full length of the MLC.

We established confidence in MPC, DailyQA3, and Delta4 by the combination of in‐house testing, review of the initial 3 years of QC results data, and literature review. MPC has been validated against conventional QC testing.^1^, ^2^, ^3^, ^4^, ^5^, ^6^, ^7^ The DailyQA3 has been validated for the measurement of output constancy, flatness, and symmetry.^16^ The Delta4 has been validated for patient‐specific QC,^17^ and its sensitivity to detecting machine errors has been demonstrated.^18^

In addition to MPC, DailyQA3, and Delta4, we perform a small set of critical conventional QC tests monthly, where test parameters do not overlap with the testing via MPC, DailyQA3, and Delta4. Further conventional QC testing is carried out at a reduced frequency, either quarterly or annually. We used our QC records of the last 3 years on eight TrueBeam Linacs to inform the reduction of test frequency, retaining QC tests monthly either if the test parameter demonstrated some instability over this time, or if it were not measured by the MPC, DailyQA3, or Delta4.

Our Linacs were clinical from November 2016, with conventional QC performed for the first 3 years, and then the hybrid QC program was introduced in January 2020. The Linac fleet comprised seven TrueBeams and one TrueBeam STx. Each Linac performs all treatment types, with the exception of stereotactic body radiotherapy (SBRT), which is performed on the TrueBeam STx only. All Linacs are equipped with the aS1200 portal imager, with the TrueBeams equipped with Millennium 120 MLC, and the TrueBeam STx with the HD120 MLC.

### 2.2 FMEA

**FIGURE 1:**
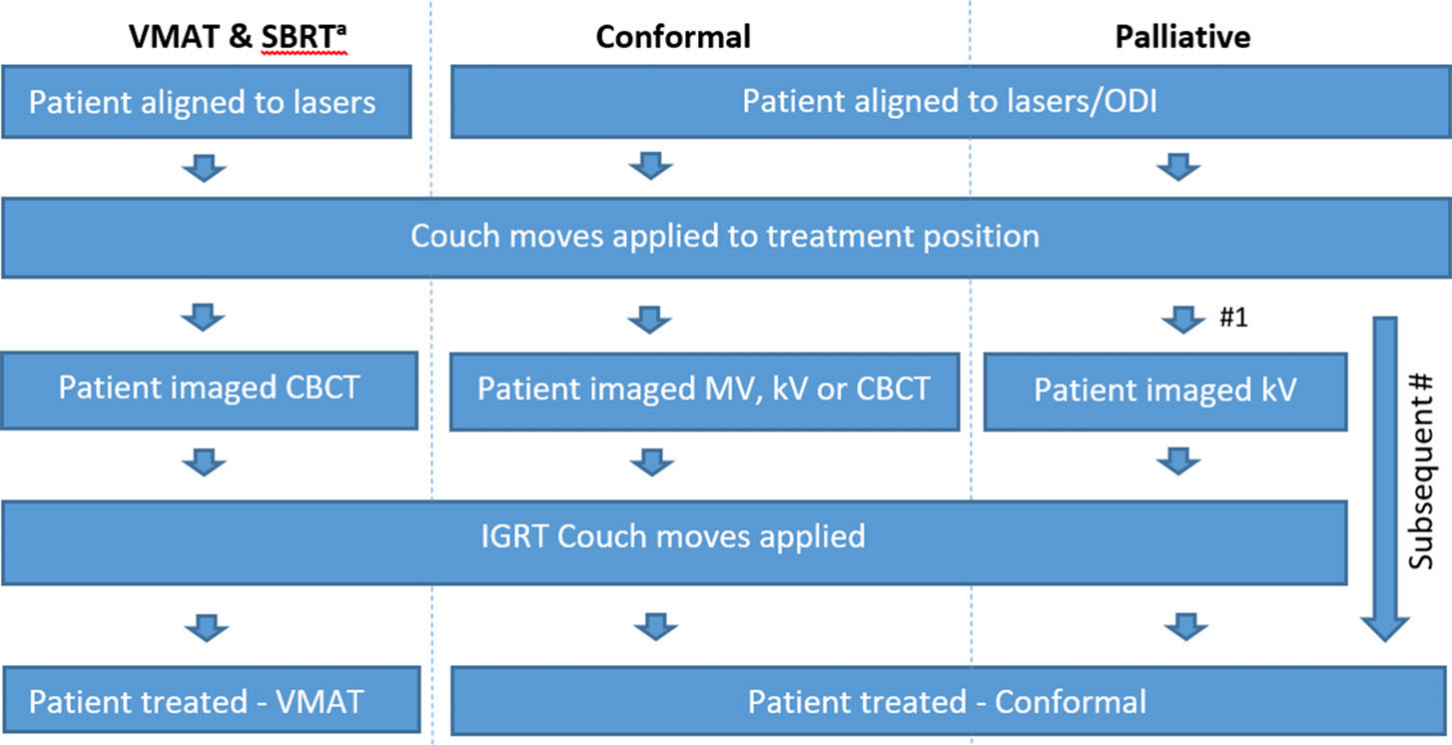
Process maps—volumetric modulated arc therapy (VMAT) and stereotactic body radiotherapy (SBRT), conformal and palliative treatments. “a” indicates that VMAT and SBRT treatments follow the same process.

An FMEA was performed in order to validate the hybrid QC program prior to implementation in January 2020. The FMEA was designed to be relevant to the particular patient cohorts and treatment types delivered on our Linacs and followed the FMEA method outlined in TG‐100. As we required the FMEA to be specific to the ways in which the Linacs are utilized for patient treatment, we analyzed the treatment pathway types in clinical use in our department. VMAT is used on the full range of treatments sites, with SBRT also utilizing VMAT treatment delivery for treating lung and oligometastases. Both pathways image the patient daily with CBCT with daily online corrections. In addition to VMAT treatments, we also deliver treatments with static conformal MLC‐shaped fields, the majority of which are breast treatments, alongside various palliative treatments. We perform daily imaging for breast and other conformal treatments, whereas palliative patients are imaged only on the first fraction. From this, we grouped the pathways into four main treatment types: VMAT, SBRT, conformal, and palliative treatments. Subsequently, a process map was created for each treatment type delivered in the department, Figure 1. These process maps outline each of the stages of the patient treatment on the Linac. They were then evaluated to identify the possible failure modes at each stage of the process for each treatment type and then identify the cause of each failure mode. We established that the root cause of an error in the patient treatment would either be an error in the patient position prior to treatment, or a machine error during the treatment delivery.

Taking the VMAT treatment type as an example, the key stages in Figure 1 are the IGRT couch moves post CBCT and the VMAT treatment. We identified the failure modes to be “patient in the wrong place post IGRT,” alongside the failure modes attributed to either beam delivery faults, mechanical faults, or VMAT delivery faults, as listed in the first column of Table 2. Then the possible specific Linac faults contributing to each of these failure modes was then identified. The specific faults resulting in the patient being in the wrong position post IGRT were errors in the treatment‐imaging isocenter, the couch move and rotation calibration, and the image match and couch repositioning process. In the same way, specific faults causing each of the beam delivery, mechanical, and VMAT delivery failure modes were identified, listed in column 2 of Table 2.

**TABLE 2:** Example of failure mode identification and severity and detectability scoring for the volumetric modulated arc therapy (VMAT) treatment type

Failure mode identification		Severity		Severity scoring, from physicists:					Detectability		Detectability scoring, from physicists:				
Failure mode	Specific Linac fault	Fault magnitude	Fault simulation method	1	2	3	4	5	Relevant QC test from hybrid QC program	QC test frequency	1	2	3	4	5
Patient in wrong place post IGRT	Treatment‐imaging isocenter	1.5 mm	Isocenter translation	7	7	7	8	8	MPC	Daily	2	3	3	3	2
	Couch moves	1.5 mm	Isocenter translation	7	7	7	8	8	MPC	Daily	2	3	3	3	2
	Couch angle	1°	Couch rotation	2	3	3	3	4	MPC	Daily	2	3	4	3	2
	Image match/couch reposition	2.5 mm	Isocenter translation	7	8	8	8	8	2D–2D match	Daily	2	3	3	3	2
Beam delivery fault	Output	3.5%	Plan renormalization	7	7	8	7	7	MPC/DailyQA3/output	Daily/weekly/monthly	2	3	3	3	2
	Beam symmetry	3.5%	Plan renormalization and literature	5	7	4	6	4	MPC/DailyQA3	Daily/weekly	4	3	4	5	3
	Beam energy	1.5%	Beam model PDDs edited	3	4	3	5	4	MPC/DailyQA3	Daily/weekly	4	3	4	5	5
	Beam position	1.5 mm	Isocenter translation	7	7	7	8	8	MPC/DailyQA3	Daily/weekly	4	3	4	5	3
Mechanical fault	Collimator angle	1°	Collimator rotation	5	4	4	5	5	MPC	Daily	2	3	3	3	2
	Gantry angle	1°	Gantry angle rotation	2	3	3	5	3	MPC	Daily	2	3	3	3	2
	Radiation isocenter	1.5 mm	Isocenter translation	7	7	7	8	8	MPC	Daily	2	3	3	3	2
VMAT delivery fault	Dose rate	1.5%	Plan renormalization	5	5	3	6	4	MPC	Daily	4	3	3	2	3
	MU1 fails to terminate	2.5%	Plan renormalization on final control point	6	6	6	8	6	MPC/DailyQA3	Daily/weekly	2	3	1	2	2
	Output with gantry	2.5%	Plan renormalization	6	6	6	6	6	VMAT standard plan	Monthly	7	4	5	4	4
	MLC position dynamic	1.5 mm	DLG increased	7	7	8	8	8	Static: MPC Dynamic: VMAT picket fence/Sweeping gap/VMAT standard plan	Daily Monthly	7	4	5	5	6
	Jaw position dynamic	2.5 mm	X‐jaw size increased	2	3	3	3	3	Static: MPC Dynamic: VMAT standard plan	Daily Monthly	7	4	4	5	6

*Note*: For each Linac failure mode identified, the fault magnitude is estimated as 0.5 mm or 0.5% above the QC tolerance. The severity scoring by Physicists 1–5 was determined from simulations of each fault in the treatment planning system, through evaluating each plan against local OAR tolerances. The detectability scoring by Physicists 1–5 was determined through identifying the particular QC from the hybrid QC program that should detect the fault and the frequency of each test.

Abbreviations: MPC, machine performance check; OAR, organ at risk; QC, quality control.

This analysis was repeated for each of the treatment types, with different failure modes becoming relevant for each of the treatment types. For example, for conformal treatments, we included potential failure modes specific to conformal delivery: errors in the jaw and MLC static position and the wedged field delivery. For palliative treatments, we image on the first fraction only and, therefore, included failure modes relating to the setup of the patient without IGRT on subsequent fractions. Here, errors in the lasers or optical distance indicator (ODI) now become relevant because there is no imaging which would correct the patient setup. In this way, we determined all the relevant failure modes for the FMEA scoring.

#### 2.2.1 FMEA scoring

In general, for each Linac fault that was identified as a possible failure mode, there was a corresponding routine QC test. The magnitude of each Linac fault condition was estimated using the conventional QC tolerance as a representative value. As reported in previous work,^11^, ^12^ we observed that QC failures were of small magnitude, at the level of QC tolerances, rather than of larger magnitudes. The scoring table published by Bonfantini et al.^12^ was used for estimating the occurrence (O), severity (S), and lack of detectability (D) of each failure mode.

#### 2.2.2 Occurrence estimates

The occurrence scores were estimated from our QC records from our fleet of eight TrueBeam Linacs over 3 years, recorded in MPC, DailyQA3, and conventional QC QATrack+ v0.2.9.1 databases (Multi Leaf Consulting, Ottawa, Canada). The frequency required for the occurrence estimate is the failure rate if the fault were not being detected and corrected. The failure rate was taken as the fault detection rate, corresponding to the frequency of each out‐of‐tolerance QC test result, excluding test results that were performed during repairs as part of the procedure to correct a fault. This detection rate was taken from our conventional QC results that were acquired prior to implementing the hybrid QC program, in the period from November 2016 until January 2020. All tests were performed according to frequencies outlined in TG‐142.^14^ The majority of QC suspend tolerances were taken from TG‐142, with the exceptions of the tests outlined in Table 3, where the tolerances come from IPEM Report 81^19^ and Report 81v2.

**TABLE 3:** Quality control (QC) test suspend tolerances followed TG‐142, except for the tabulated tests where the tolerance was taken from Report 81v2

Frequency	Test	Suspend tolerance
Daily	Laser localization	2 mm
	ODI	3 mm
Monthly	Light field jaw size	1 or 2 mm^a^
	Gantry/collimator angle indicators	0.5°
	Flatness and symmetry	3%^b^
	Output with dose rate	1%
Annual	Output constancy vs. gantry angle	2%^b^

^a^1 mm for jaw setting of ≤15 cm and 2 mm for 20 cm.

^b^Tolerance taken from original version of Report 81.

The occurrence estimate required the failure rate if the particular parameter were not monitored and corrected. This is the case for the majority of faults that occur randomly without any specific underlying cause, with the exception of output. Output is observed to systematically increase over time on all of our Linacs and is routinely adjusted down before results are out of tolerance. Our local data demonstrated an increase in the output of between 3% and 4% per year for our eight Linacs over the first 3 years of Linac use.^7^ Therefore, outputs are monitored monthly with chamber measurements and adjusted if greater than 1.5% from 1 cGy/MU in order to maintain the output at a stable level. We have not changed this process in the hybrid QC program. Therefore, it is suitable to take the occurrence estimate for the output failure mode in the same way as outlined earlier for the other faults, that is, the detection rate based on our conventional QC results. It represents the failure rate assuming the output is being monitored and corrected if greater than 1.5% than expected.

There were two Linac fault conditions identified where we did not carry out a specific QC test: dynamic MLC and dynamic Jaw failures. Here, we made the occurrence estimation from the closest test from our QC program, that of the MLC picket test and static jaw test, respectively. This estimate was justified as in each of these reported static failures, the dynamic delivery would equally have been impacted.

#### 2.2.3 Severity estimates

We made quantitative estimates of the severity of each identified failure mode for each of the treatment types by simulating faults in our Eclipse treatment planning system (version 15.1, Varian Medical Systems, Inc., Palo Alto, CA, USA). The faults were simulated at either 0.5 mm or 0.5% above suspend QC tolerance, with them affecting all treatment fractions for a worst case scenario. In order that the FMEA cover the most conservative option, we selected a representative treatment site for each treatment type that was expected to give the worst case scenario, which is the highest severity estimate based on the proximity of organ at risk (OAR) to treatment volume and OAR dose constraints. The treatment sites for each treatment type were head and neck for VMAT, spine metastasis for SBRT, tangential breast for conformal, and cord compression/stomach for palliative. Three plans were selected for each representative treatment site (Table 4). The plans were then recalculated with a simulated error for each failure mode using fixed monitor units (MU) (except for when simulating the error in output) with the clinical algorithm originally used to create the plan (Acuros for VMAT and SBRT, AAA for conformal and palliative). Faults that would result in an error in the patient position relative to the isocenter prior to treatment (treatment‐imaging isocenter, radiation isocenter, couch position, beam position, laser position, and light field crosshair position) were simulated by the movement of the treatment isocenter in the six translational directions, with the worst result reported. Similarly, couch and collimator angle faults were simulated by adjusting each rotation and reporting the worst direction fault. Gantry angle and jaw size faults were simulated by increasing the gantry angle and jaw size at each control point for each beam. High and low output faults were simulated by the renormalization of the plan to increase or decrease the MU, respectively. The dynamic MLC position error was simulated by increasing the dosimetric leaf gap parameter within the planning system, and a wedge fault was simulated by increasing the number of MU through the wedged fields. The beam energy fault was simulated by inputting new percentage depth–dose curves into the Eclipse beam model that had been edited to exhibit a higher energy. We were unable to simulate errors in symmetry because on attempting to input asymmetric beam data into Eclipse, it was rejected by the beam modeling software due to the asymmetry. Therefore, the likely effect of asymmetry was inferred by assessing the simulated errors for output, alongside reviewing the severity estimates for symmetry faults from Bonfantini et al.^12^

**TABLE 4:** Treatment plans used for the error simulations in the treatment planning system

Treatment type	Treatment site	Fractionation	Description
VMAT	Head and neck	65/54 Gy in 30# 65/54 Gy in 30# 70/56 Gy in 35#	Nasopharynx Oropharynx Oropharynx and neck
SBRT	Spine metastasis	27 Gy in 3# 30 Gy in 6# 30 Gy in 3#	C7, mid‐heart height C7, top of PTV in line with top of heart Rib lesion, abutting C8, height of right kidney and large bowel
Conformal	Tangential breast	40 Gy in 15# 40 Gy in 15# 40 Gy in 15#	Left Sided, heart shielding Left sided, supraclavicular node Left sided, internal mammary chain, supraclavicular lymph node, heart shielding
Palliative	Cord compression/stomach	20 Gy in 5# 15 Gy in 5# 8 Gy in 1#	Cord compression, T4/T5, single field Cord compression, T2–T7, ant‐post fields Stomach, ant‐post fields with MLC shaping

Abbreviations: PTV, planning target volume; SBRT, stereotactic body radiotherapy; VMAT, volumetric modulated arc therapy.

The severity scores were estimated by averaging the independent survey results of five medical physicists from our center, each with between 14 and 17 years of experience in radiotherapy. Scorers were presented with the original and modified clinical plans and then assessed them against local clinical protocol OAR tolerances and ICRU 83^20^ guidance on planning target volume (PTV) coverage. The severity of each failure mode was then scored following the table in Bonfantini et al.^12^

##### Example: severity estimates for VMAT

We have included the scoring process for severity as applied to VMAT treatments as an example (Table 2). For each Linac fault as determined from the analysis of the process maps, the fault magnitude was estimated as 0.5 mm or 0.5% above suspend QC tolerance. Each of the three head‐and‐neck plans (with PTVs in the nasopharynx, oropharynx, and neck) was recalculated with each failure mode simulated in Eclipse, and the effect on OAR doses and PTV coverage was assessed. Taking output as an example, increasing the output by 3.5% caused the OAR dose limits to be exceeded: For the nasopharynx patient, the maximum optic chiasm and optic nerve, and mean larynx dose tolerances were exceeded by 0.5 Gy. For the oropharynx patient, the spinal cord and spinal cord planning risk volume (PRV) maximum dose and D0.1cc dose constraints were exceeded, whereas the dose to the PTV increased such that *D*5% was greater than 105%. For the oropharynx and neck patient, the spinal cord PRV maximum dose was exceeded, increasing from 48.4 to 50.1 Gy. This severity was consequently scored as 7 or 8 by survey participants. Then, taking gantry angle as an example, simulating a 1° error for each of the three patients, there were minimal differences in the PTV coverage and OAR doses, with all OAR remaining within tolerance, resulting in severity scores ranging from 2 to 5.

#### 2.2.4 Detectability estimates

The detectability scoring estimated the probability of a fault going undetected over a typical treatment course, assuming that routine Linac QC was being performed according to the hybrid QC program (Table 1). The treatment course length was taken as a month for conventional and VMAT radiotherapy treatments, and a week for SBRT and palliative treatments. As with the severity scoring, the detectability scores were estimated by averaging each of the estimates from the independent survey results of the five medical physicists from our center, following the scoring table in Bonfantini et al.^12^

We have evaluated the accuracy of MPC in detecting QC failures as part of the commissioning process of MPC in our center. This previous work^7^ assessed the effectiveness of MPC in detecting real‐world QC faults over the 3‐year period by comparison with conventional QC testing records, through the analysis of the true negative and false negative out of tolerance MPC results, taking conventional QC as the ground truth. This work demonstrated the accuracy of MPC with respect to conventional QC, showing MPC is capable of detecting beam and mechanical faults at a level that is appropriate for QC. This was in keeping with other authors, who report the results of sensitivity testing to deliberate errors in mechanical and imaging parameters, where MPC demonstrated sub‐mm and sub‐degree accuracies.^2^, ^3^, ^4^, ^6^ Our previous publication^7^ identified that the exception to this high level of accuracy was the user‐calibrated aspect of MPC, the Beam group, which comprises output, uniformity, and center shift test parameters. Due to the processes around baselining the output, there will inherently be some uncertainty between the results of these test parameters as compared to the conventional QC results. This is also the case for the test parameters measured by the DailyQA3. We accounted for this effect in the detectability scoring by performing an uncertainty analysis (Table 5). The maximum uncertainty in the reported MPC or DailyQA3 parameter was determined through comparison with conventional QC results and tolerances.

**TABLE 5:** Uncertainty analysis of machine performance check (MPC) and DailyQA3 test parameters

QC tool	Test parameter	Tolerance	Maximum uncertainty
MPC beam group	Output	2%	1.5%
	Uniformity	2%	2%
	Centre shift	0.5 mm	1 mm
DailyQA3	Output	3%	1.5%
	Flatness	3%	2%
	Symmetry	3%	1%
	Centre shift	2 mm	1 mm
	Field size	3 mm	1 mm

*Note*: Suspend level tolerance and maximum uncertainty in reported test result for the MPC Beam group and DailyQA3.

Abbreviation: QC, quality control.

The process of estimating the detectability of each fault was as follows:

1. The physicists took each specific Linac fault in turn and identified the QC from the hybrid QC program that might detect it by referring to Table 1, which outlines the test frequencies and tolerances.
2. They reviewed the magnitude of the fault relative to the tolerances set in the particular QC test that should identify the fault. They referred to Table 1, as well as tables of the individual test parameters tolerances for the automated QC elements (for MPC this is summarized in Table 1 in Pearson et al.^7^).
3. For the automated QC tests where uncertainty in the reported parameter had been identified (MPC Beam group or DailyQA3), they also referred to the uncertainty analysis in Table 5 to establish if the fault would be detected.
4. Assuming the particular fault level was greater than the test tolerance level and uncertainty if applicable, and therefore detectable, the major contributing factor to the detectability estimate was the frequency of the QC test performed which should pick up the fault, resulting in low detectability estimates for any faults where the relevant QC test is performed daily. Survey participants assessed the frequency of the QC test and, with reference to the treatment course length, estimated the probability of a fault going undetected over a typical treatment course.

Additional factors that feed into the estimate are as follows:

- If there is redundancy in QC checks, for example, between MPC and DailyQA3, there is overlap between the different tests performed, and therefore, increased likelihood of an issue being detected.
- If not directly tested by a particular check, whether the parameter may be detected indirectly via another test that is performed.

##### Example: detectability estimates for VMAT

We have included the scoring process for detectability as applied to VMAT treatments as an example (Table 2). For each Linac fault as determined from the analysis of the process maps, the QC test that should detect the fault has been selected from the hybrid QC program. The frequency of each of these tests is presented and was used to inform the independent detectability scoring by the five medical physicists from our center. Some estimates were straightforward. Taking output as example, and assuming a fault level of 3.5%, this should be detected by the daily MPC measurement (suspend tolerance 2%, maximum uncertainty 1.5%) and is reasonably likely to be detected by the weekly DailyQA3 measurement (suspend tolerance 3%, maximum uncertainty 1.5%). The calibration of both the devices is tested monthly versus Farmer ionization chamber measurements with a 1% warning tolerance, and 1.5% suspend tolerance. Consequently, the probability of the failure going undetected over the course of treatment was classed as 0.2% or 0.5% by survey participants, generating scores 2 or 3. Survey participants chose not to score as 1, a probability of the fault going undetected as <0.01%, as there is some potential for an output of 3.5% not being detected, if there were drift between both MPC, DailyQA3 and the Farmer ionization chamber output. Other detectability estimates were less straightforward, taking the MLC position fault during dynamic treatment as an example, assuming a fault level of 1.5 mm, it is less clear that this fault would be picked up by the hybrid QC program. The daily MPC measurement only checks the MLC in a single static position at gantry angle 0°, and MLC is not checked via the DailyQA3 measurements where jaws are used to define the field size. If not detected by MPC, the detection of the fault would then rely on it being picked up during the monthly checks of sweeping gap, VMAT picket fence, or the VMAT standard plan. Each of these tests may detect the fault depending on the way in which the fault is manifested. For example, the fault could be detected by sweeping gap if it involved central leaves, but this is less likely for outer leaves where the impact on the change in charge measured by the centrally poisoned ionization chamber would be minimal. All these factors feed into the each person's estimate; consequently, the scoring is more subjective, and the spread of estimates between survey participants may be greater.

#### 2.2.5 Risk priority number

The RPN was calculated from the product of the O, S, and D indices and then normalized to the highest RPN value. Failure modes were ranked according to the normalized RPN to indicate the most critical failure modes.

### 2.3 Comparison of QC faults before and after implementation of hybrid QC

We reviewed all our QC records, from November 2016, when the Linacs were put into clinical service, through January 2020 when the hybrid QC program was introduced, until August 2021, thus giving just over 3 years of data from the conventional QC program, and 1.5 years of data from the hybrid QC program. We were then able to compare the QC faults that measured greater than the suspend level tolerance, in the period in which the conventional QC was performed, versus the period when the hybrid QC program was in place.

## 3 RESULTS

### 3.1 FMEA results

The failure modes for each treatment type have been identified and the FMEA scores estimated for the application of the hybrid QC program at our center. The O, S, D, and RPN scores are in Table 6. There was close overall agreement between the estimates of the severity and detectability by each of the five medical physicists, with mean ranges of 2 for both severity and detectability scoring.

**TABLE 6:** Failure mode and effects analysis (FMEA) scores for each treatment type

Treatment type	Failure mode	Specific Linac fault	Magnitude	Occurrence	Severity	Detectability	RPN	Normalized RPN
VMAT	Patient in wrong place post IGRT	Treatment‐imaging isocenter	1.5 mm	2	7.4	2.6	38	23
		Couch moves	1.5 mm	1	7.4	2.6	19	12
		Couch angle	1°	1	3	2.8	8	5
		Image match/couch reposition	2.5 mm	1	7.8	2.6	20	12
	Beam delivery fault	Output	3.5%	1	7.2	2.6	19	12
		Beam symmetry	3.5%	2	5.2	3.8	40	24
		Beam energy	1.5%	1	3.8	4.2	16	10
		Beam position	1.5 mm	2	7.4	3.8	56	34
	Mechanical fault	Collimator angle	1°	1	4.6	2.6	12	7
		Gantry angle	1°	1	3.2	2.6	8	5
		Radiation isocenter	1.5 mm	1	7.4	2.6	19	12
	VMAT delivery fault	Dose rate	1.5%	1	4.6	3.0	14	9
		MU1 fails to terminate	2.5%	1	6.4	2.0	13	8
		Output with gantry	2.5%	1	6	4.8	29	18
		MLC position dynamic^a^	1.5 mm	4	7.6	5.4	164	100
		Jaw position dynamic^a^	2.5 mm	1	2.8	5.2	15	9
SBRT	Patient in wrong place post IGRT	Treatment‐imaging isocenter	1.5 mm	2	8	2.6	42	26
		Couch moves	1.5 mm	1	8	2.6	21	13
		Couch angle	1°	1	4.6	2.8	13	8
		Image match/couch reposition	2.5 mm	1	9	2.6	23	14
	Beam delivery fault	Output	3.5%	1	7	3.0	21	13
		Beam symmetry	3.5%	2	5.8	3.6	42	26
		Beam energy	1.5%	1	6.2	3.8	24	15
		Beam position	1.5 mm	2	8	3.6	58	35
	Mechanical fault	Collimator angle	1°	1	4	2.6	10	6
		Gantry angle	1°	1	3.4	2.6	9	5
		Radiation isocenter	1.5 mm	1	8	2.6	21	13
	VMAT delivery fault	Dose rate	1.5%	1	5.8	3.0	17	10
		MU1 fails to terminate	2.5%	1	6.4	2.2	14	9
		Output with gantry	2.5%	1	6	4.4	26	16
		MLC position dynamic^a^	1.5 mm	4	7.4	5.0	148	90
		Jaw position dynamic^a^	2.5 mm	1	3.2	5.0	16	10
Conformal	Patient in wrong place post IGRT	Treatment‐imaging isocenter	1.5 mm	2	4.6	2.6	24	15
		Couch moves	1.5 mm	1	4.6	2.6	12	7
		Couch angle	1°	1	3.8	2.8	11	7
		Image match/couch reposition	2.5 mm	1	5.5	2.6	14	9
	Beam delivery fault	Output	3.5%	1	7.2	2.6	19	12
		Beam symmetry	3.5%	2	6.2	3.8	47	29
		Beam energy	1.5%	1	4.4	4.2	18	11
		Beam position	1.5 mm	2	4.6	3.8	35	21
		Wedge	2.5%	1	6.4	4.6	29	18
	Mechanical fault	Collimator angle	1°	1	5.9	2.6	15	9
		Gantry angle	1°	1	4	2.6	10	6
		Radiation isocenter	1.5 mm	1	4.6	2.6	12	7
		MLC position static	1.5 mm	4	3.2	3.0	38	23
		Jaw position static	2.5 mm	1	6.6	3.0	20	12
Palliative	Patient in wrong place post alignment	Laser position	2.5 mm	1	5.2	2.8	15	9
		ODI	3.5 mm	1	5.4	3.2	17	10
		light field cross hair	1.5 mm	1	5.2	4.4	23	14
		Couch moves	1.5 mm	1	5.2	3.0	16	10
		Couch angle	1°	1	2.4	3.2	8	5
		Couch deflection	3.5 mm	1	5.4	5.8	31	19
	Beam delivery fault	Output	3.5%	1	6.4	3.0	19	12
		Beam symmetry	3.5%	2	4.8	3.6	35	21
		Beam energy	1.5%	1	4	3.8	15	9
		Beam position	1.5 mm	2	5.2	3.6	37	23
		Wedge	2.5%	1	6.2	5.8	36	22
	Mechanical fault	Collimator angle	1°	1	2.2	2.6	6	4
		Gantry angle	1°	1	2.2	2.6	6	4
		Radiation isocenter	1.5 mm	1	5.2	2.6	14	9
		MLC position static	1.5 mm	4	2.2	3.0	26	16
		Jaw position static	2.5 mm	1	5.2	3.3	17	10

Abbreviations: RPN, risk priority number; SBRT, stereotactic body radiotherapy; VMAT, volumetric modulated arc therapy.

^a^We did not carry out a specific QC test for MLC or Jaw dynamic movement. Therefore we made the occurrence estimation from the closest tests from our QC program: MLC picket test and static jaw test. This estimate was justified as each of these reported static failures would have impacted on dynamic delivery.

The highest normalized RPN values indicate that the most critical Linac failure mode was MLC position dynamic, for both VMAT and SBRT treatments, with scores of 100 and 90. After this, the next highest RPN is 35 for the beam position failure mode for SBRT treatments. The majority of failure modes have low normalized RPN values, with 19% in the range 35–20 and 77% less than 20.

### 3.2 Comparison of QC faults before and after implementation of hybrid QC

All the QC test failures that measured greater than the suspend level tolerance are summarized in Table 7. There were a total of 10 failures, with 7 in the 3 years prior to the implementation of the hybrid QC program in January 2020, and 3 over the following 1.5 years.

**TABLE 7:** Table of quality control (QC) test failures, measured greater than the suspend level tolerance

Time period	Test	Suspend tolerance	Linac	Date	QC result
November 2016–January 2020	Isocenter verification	1 mm	G5	01/07/2020	1.7 mm
	Beam symmetry	3%	G4	06/13/2019	3.03%
	Radiation field size	2 mm	G2	05/03/2019	2.5 mm
	MLC position	1 mm	G2	10/07/2017	1.7 mm
				06/05/2019	1.4 mm
			G5	09/28/2018	1.1 mm
				09/18/2019	2.0 mm
January 2020–August 2021	Lasers v optical‐mechanical isocenter	1 mm	G2	05/14/2020	1.1 mm
	Light field Jaw size	2 mm	G5	03/05/2021	3.0 mm
	MLC position	1 mm	G2	02/14/2020	1.1 mm

*Note*: The hybrid QC program was implemented in January 2020. The table includes test failures before and after the implementation of hybrid QC.

## 4 DISCUSSION

We have applied FMEA to confirm the validity of the hybrid QC schedule at our center, with the FMEA specific to the treatment types delivered on our Linacs. This follows the approach established by O'Daniel et al.^11^ who applied FMEA specific to their clinic, and Bonfantini et al.^12^ who applied FMEA specific to individual Linacs in their clinic. We have made this FMEA more focused still, making it specific to the delivery of each clinical treatment type at our center by identifying relevant failure modes for each step in the specific treatment type process map (VMAT, SBRT, conformal, palliative).

Additionally, rather than applying FMEA to an already established QC program, to determine a ranked priority of tests,^10^, ^11^, ^12^ this is the first application of FMEA to validate a new QC program. We applied FMEA to the novel hybrid QC program as part of the validation process prior to implementation. Instead of considering all standard QC tests as possible failure modes, as done by O'Daniel et al.^11^ for the daily QC tests in TG‐142^14^ and by both Smith et al.^10^ and Bonfantini et al.^12^ for all tests in TG‐142,^14^ we considered which particular Linac faults could occur as failure modes at each stage in the treatment process maps (Figure 1). This established the failure mode as the Linac failure and not the QC test failure, in a different approach to FMEA as has been previously applied to Linac QC.^10^, ^11^, ^12^

This difference in approach further impacted our detectability scoring process as compared with previous work. O'Daniel et al.^11^ ranked detectability as 10 for all tests, assuming that any error associated with each daily QC test would be impossible to detect if the test itself were not performed. The weakness in this method is the RPN estimate consequently omits any difference in detectability. Smith et al.^10^ and Bonfantini et al.^12^ estimated the detectability of each test parameter by assuming that if the particular test were not performed, what would be the likelihood that the fault would be picked up by another pathway, such as from a different QC test, or via machine interlocks. Our detectability scoring took a different starting point: For each Linac fault failure mode, the detectability was the probability that the testing according to the hybrid QC program would detect the particular fault. Consequently, this FMEA determines if the hybrid QC test program is acceptable and able to detect all Linac failure modes, and this process will highlight if there are any omissions from the hybrid QC program. This assumed that the individual components of the hybrid QC such as MPC were functioning correctly. We had previously validated MPC at our center^7^ by comparing its ability to detect real‐world faults with conventional QC, and it has shown to correctly detect deliberate errors.^2^, ^3^, ^4^, ^6^

The previous application of FMEA to Linac QC has involved a variety of scoring methods, from survey based^10^ on quantitative methods.^11^, ^12^ By using our QC records of failures on our Linacs, we were able to make the occurrence scores quantitative and specific to the Linac fleet at our center. These data are equivalent to 24 Linac years and may be a useful resource for other centers with the same Linac model and similar QC tolerance levels. Similarly, the severity scoring was based on the analysis of a sample of our treatment plans for each treatment type delivered on our Linacs, ensuring that the FMEA is relevant to our center. The severity and detectability scoring via surveys showed close agreement among physicists in our department, and there is generally better agreement than reported by Bonfantini et al.^12^ The mean range in our estimates for each severity and detectability score was 2 for both, compared with 4 and 5 for their Varian TrueBeam. This may be due to the higher and more similar level of experience of all our survey participants in both Linac QC and treatment planning. In addition, as stated earlier, the detectability scoring by Bonfantini et al.^12^ estimated detectability if the particular test were not performed, whereas we estimated the detectability of each failure mode assuming the hybrid QC schedule were in place.

We are able to compare our results versus previous applications of FMEA to Linac QC^10^, ^11^, ^12^ by comparing relative RPN values, where the RPN values are normalized to the highest scoring RPN. Our distribution of the relative RPN scores is similar to the distribution reported by Bonfantini et al.^12^ Each shows a few failure modes with high scores, based on VMAT delivery, then the normalized RPN scores tend to quickly fall, with most scores less than 20. Output was reported to be the highest RPN by both Smith et al.^10^ and O'Daniel et al.,^11^ whereas Bonfantini et al.^12^ reported it as the highest RPN for their Varian TrueBeam, with output ranking third on their other two Linacs. However, we observe low RPN for our output failure mode due to the low occurrence score, a consequence of regular monthly monitoring and adjustment if greater than 1.5% from nominal values.

Our highest relative RPN is for the “MLC position dynamic” for VMAT and SBRT (Table 6). This indicates a potential weakness in the hybrid QC program as proposed in Table 1. The MLC testing consists of MPC MLC positional accuracy, sweeping gap, VMAT MLC Picket Fence, and VMAT Delta4 Standard Plan, but there is no specific testing of dynamic MLC. The fact that the FMEA highlighted this weakness has demonstrated the value of performing the FMEA. This is potentially something that may have been overlooked if the FMEA had not been carried out. We are in the process of incorporating specific dynamic MLC testing in order to make the hybrid QC program more robust. Similarly, this is also a high scoring failure mode for Bonfantini et al.,^12^ named, “MLC speed (VMAT).” Ideally, Linac manufacturers will incorporate dynamic MLC testing into future versions of the automated QC tools, which would be particularly valuable as a high proportion of clinical treatments on modern Linacs involve VMAT. This would be a welcome addition to the current MPC testing sequence.

Both O'Daniel et al.^11^ and Bonfantini et al.^12^ utilized FMEA to identify the most critical tests and have optimized the QC schedule by reducing the frequency of the tests with the lowest RPN scores. O'Daniel et al.^11^ applied this to daily tests, whereas Bonfantini et al.^12^ reduced the frequency of the MU linearity, X‐ray output constancy versus dose rate, X‐ray beam quality, and X‐ray flatness and symmetry monthly tests to annual. This work confirms that Linac QC can be optimized to increase efficiency and maintain safety and treatment quality. Through evaluating the normalized RPN values, we have determined that the hybrid QC schedule outlined in Table 1 is acceptable. Each of the fault modes after “MLC position dynamic” has low relative RPN values, validating the testing parameters and frequencies set out in hybrid QC. It should be noted, however, that these FMEA results are specific to our center, and we would advise that any center intending on adopting a hybrid QC program should carry out an FMEA locally, as recommended in TG‐100^13^ and AAPM Medical Physics Practice Guideline 8.a.^10^

The comparison of Linac faults before and after implementation of the hybrid QC program, Table 7, enables some assessment of the introduction of the hybrid QC program. There are few faults, a total of 10 for the 8 Linacs over 4.5 years, equating ∼1 fault per Linac every 4 years, demonstrating the inherent stability of the TrueBeam Linacs. There is a similar frequency of events before and after the implementation of hybrid QC, with seven events in the 3 years before implementation, and three events in the 1.5 years of hybrid QC. The magnitude of the faults is similar before and after implementation, at a level just above the QC tolerances as previously observed in the literature.^11^, ^12^ There is no increase in the frequency or magnitude of QC faults on the introduction of hybrid QC, supporting our implementation of this new QC program.

Our QC program is continually evolving in our department, with the aim of automating the test procedures and analysis of the conventional tests we perform. We have automated the calculations and image analysis for some tests with QATrack+ and Pylinac (//github.com/jrkerns/pylinac) Python scripts. The weakness in our MLC QC as highlighted by the FMEA led us to implement this analysis for VMAT MLC Picket Fence. We have moved from analysis via the visual inspection of the image to automated analysis of the deviations of each individual picketed versus the picket average position. This has allowed us to implement a much tighter tolerances than visual inspection, with a suspend level tolerance of 0.2 mm on individual MLC picket position, giving us greater confidence in our MLC delivery during VMAT. We are developing further testing of MLC, and as MPC continues to be developed by Varian, we intend to keep the hybrid QC program under constant review. As new test parameters are added and validated, we expect to utilize MPC for these routine tests while continuing to perform the equivalent conventional QC at reduced frequency to maintain independent testing.

## 5 CONCLUSIONS

The FMEA has proved to be a useful method for evaluating the hybrid QC program and has provided confidence in implementing this new QC program at our center. This approach realizes the benefits of automated testing and enables us to make efficiency gains with regard to both medical physicist and clinical Linac time. The ranked relative RPN scores indicate that overall, the hybrid QC testing covers the critical potential fault conditions, and the test frequencies are appropriate. The high RPN score associated with the “MLC position dynamic” failure mode has usefully highlighted the requirement to incorporate regular specific dynamic MLC testing in our QC schedule.

## CONFLICT OF INTEREST

The authors have no relevant conflicts of interest to disclose.

## AUTHOR CONTRIBUTION

Michael Pearson, Victoria Butterworth, Sarah Misson‐Yates, Marium Naeem, Regina Gonzalez, David Eaton, and Tony Greener have each made substantial contributions to the acquisition, analysis, or interpretation of data for the work/and drafting the work or revising it critically for important intellectual content/ and final approval of the version to be published/ and agree to be accountable for all aspects of the work in ensuring that questions related to the accuracy or integrity of any part of the work are appropriately investigated and resolved.

## Data Availability

The data that support the findings of this study are available from the corresponding author upon reasonable request.
